# Preventive effect and safety of Chinese herbal medicine for oral mucositis during radiotherapy in patients with head and neck cancer: Study protocol for a randomized trial

**DOI:** 10.1016/j.conctc.2022.100912

**Published:** 2022-03-20

**Authors:** Yu-Chen Cheng, Yu-Ming Wang, Yun-Hsuan Lin, Jen-Yu Cheng, Shau-Hsuan Li, Yu-Chuen Huang, Ming-Yen Tsai

**Affiliations:** a Department of Chinese Medicine, Kaohsiung Chang Gung Memorial Hospital and Chang Gung University College of Medicine, Kaohsiung, 83301, Taiwan; b Department of Radiation Oncology, Kaohsiung Chang Gung Memorial Hospital and Chang Gung University College of Medicine, Kaohsiung, 83301, Taiwan; c Department of Hematology-Oncology, Kaohsiung Chang Gung Memorial Hospital and Chang Gung University College of Medicine, Kaohsiung, 83301, Taiwan; d Department of Medical Research, China Medical University Hospital and School of Chinese Medicine, China Medical University, Taichung, 41354, Taiwan

**Keywords:** Oral mucositis, Radiotherapy, Head and neck cancer, Chinese herbal medicine, Randomized trial

## Abstract

**Background:**

Oral mucositis (OM) is a common side effect of radiotherapy (RT) that can have severe implications in patients with head and neck cancer (HNC). Traditional Chinese medicine (TCM) formula is widely applied in treating OM, but little substantial evidence exists to clarify it effects. The study intends to determine whether the TCM-based prescription in treating HNC with RT can improve the OM when compared with RT alone.

**Methods:**

A single-center, randomized, two-arm parallel-group, open-label controlled clinical trial will be conducted to determine whether the Zi-Yin-Liang-Ge-San (ZYLGS), which contains *Rx. Scutellariae*, *Rx. Glycyrrhizae*, *Hb. Dendrobii*, *Rx. Ophiopogonis*, and *Hb. Menthae Haplocalycis,* combined with RT can improve the incidence and severity of OM. Two hundred participants will randomly 1:1 to receive at least 6 weeks of RT plus ZYLGS powder or control. The primary outcome measures are onset, gradation of OM (Common Terminology Criteria for Adverse Events v5.0), and oral pain (visual analogue scale). The secondary outcome measures include nutritional status, the EORTC Quality of Life Core Questionnaire and head and neck module. The Patient-Reported Outcomes version of the Common Terminology Criteria for Adverse Events, serious adverse events, and blood and biochemical analysis will be recorded to evaluate the safety. Visits will be performed for each week during the RT treatment period and then 2 weeks in the follow-up period.

**Discussion:**

The study's result will provide a high-level evidence for TCM-based formulation for HNC patients with RT on the effect of OM prevention and management.

## 1 Introduction

Oral mucositis (OM) is an iatrogenic condition characterized by erythematous inflammatory changes. It usually manifests on buccal and labial surfaces, on the underside of the tongue, and on the floor of the mouth, as well as on the soft palate of patients receiving radiotherapy (RT) [1]. The severity of OM has a wide range of severities. Some patients develop superficial sore erythema, while others present complete mucosal ulceration in the oral mucosa and pharynx. OM has multiple effects that can compromise the patient's health. Besides causing pain, it can lead to dysphagia, which can reduce nutritional intake. Mucosal ulceration can increase the risk of infection, leading to interruption of cancer treatment. All of these can collectively affect the patient's quality of life (QoL) [2,3]. OM is a frequent adverse event (AE) in patients with head and neck cancer (HNC) treated with RT and/or chemotherapy [4]. This side effect of combination therapy can reduce patients' compliance with treatment, increase the patient's economic burden, and reduce cancer survivorship [5,6]. Timely and adequate intervention for OM is very important.

The main therapeutic goals in treating patients with OM are symptom alleviation and reduction of complications [7]. To achieve these goals, various therapeutic modalities can be employed. Some examples are antioxidants, anti-inflammatory agents, antimicrobials, glutamine, colony-stimulating factors, or low-level laser. However, the efficacy of these approaches is inconsistent in the prevention or treatment of OM [8]. Therefore, adjuvant drugs capable of reducing the incidence of OM and minimizing the associated morbidity of NHC patients should be found.

Chinese herbal medicine (CHM) is a complementary and integrative medicine (CIM) for preventing or treating disease. It is based on medical plants, various minerals, and some animal parts. Having originated in East Asia, it is widely used worldwide [9]. In recent years, many patients with OM have used CHM to prevent or treat RT-induce OM [[10], [11], [12]]. Most CHMs have a “clearing heat” property that can prevent heat toxin accumulated due to RT from gradually consuming the body's energy (*qi*) and body fluid and nutrition (yin) [13], and it has an expected effect in treating OM, according to the theory of traditional Chinese medicine (TCM). The antioxidants in CHM can decrease the production of reactive oxygen species and thereby reduce the severity of mucositis [14]. To date, however, few evidence-based studies on the efficacy of TCM treatment in patients with OM have been conducted.

To explore the potential of CHM formula in preventing OM, we designed a randomized controlled trial study to examine the efficacy of CHM in preventing OM in patients with HNC.

## 2 Methods and analysis

### 2.1 Study design

**Fig. 1:**
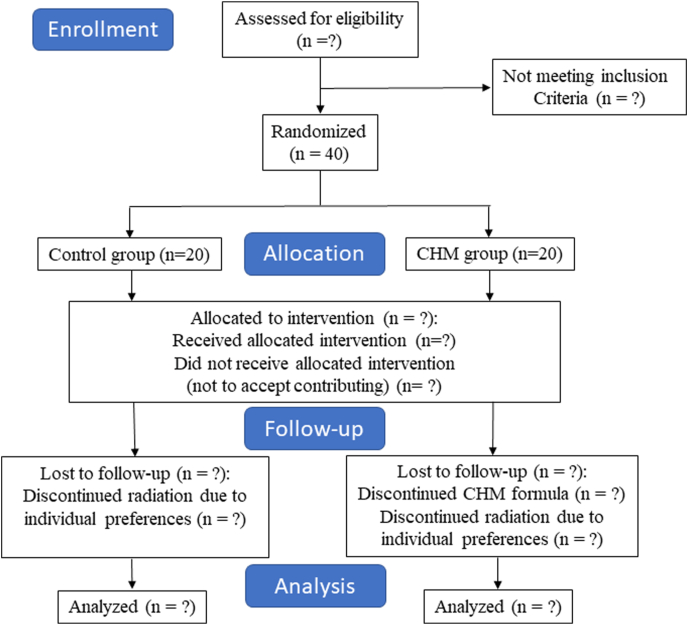
Study design from enrollment to analysis following the CONSORT 2010 flow diagram.

This study is a randomized, double-arm, parallel-group, open-label controlled clinical trial conducted at Kaohsiung Chang Gung Memorial Hospital (KCGMH), Taiwan, from August 2021 to April 2023. Patients with HNC scheduled to receive RTO as a component of their cancer treatment will be recruited. The flow chart is shown in Fig. 1. The authors will evaluate the protective effect of a TCM-based prepared medication named Zi-Yin-Liang-Ge-San (ZYLGS), which contains *Rx. Scutellariae*, *Rx. Glycyrrhizae*, *Hb. Dendrobii*, *Rx. Ophiopogonis*, and *Hb. Menthae Haplocalycis*, in preventing and managing OM in patients with HNC receiving postoperative adjuvant or definitive RT or concurrent chemo-radiotherapy therapy (CCRT) as compared with a control group. The methods will remain unchanged throughout the trial. The research protocol has been approved by the Ethics Committee of the KCGMH (202101231A3) and registered at ClinicalTrials.gov (NCT05040425). The Standard Protocol Items: Recommendation for Interventional Trials (SPIRIT) 2013 checklist is provided in **Additional file 1**.

The study is currently ongoing; the first participant was included on 12 September 2021. We expect to include the last participant in December 2022 and complete the study by April 2023.

### 2.2 Study participants

Participants will continue to be recruited from KCGMH via posters and advertisements published on the official hospital website. The recruitment period is expected to last 16 months. Informed consent will be collected from all participants before randomization.

### 2.3 Eligibility criteria

#### 2.3.1 Inclusion criteria

Participants meeting the following criteria are eligible:

1. Patients aged 20–75 years with histologically proven stage II-IV squamous HNC;
2. Indication for RT or CCRT;
3. No history of antitumor therapies;
4. No history of oral ulcer or salivary gland diseases;
5. Normal vital signs (body temperature: 36–37.5 °C; heart rate: 60 to 100 beats per minute; respiratory rate: < 20 per minute; mean arterial pressure: 70–100 mmHg), and Eastern Cooperative Oncology Group (ECOG) performance status of 0–2.

#### 2.3.2 Exclusion criteria

The exclusion criteria are as follows:

1. Terminal cancer for which aggressive treatments are unsuitable;
2. Impaired renal or hepatic function at initial diagnosis (including chronic kidney disease stages III–V and Aspartate transaminase (AST), Alanine aminotransferase (ALT) ≥ 5 × the upper normal limit);
3. Uncontrolled psychiatric problems or altered mental status;
4. Previous receipt of medications for other clinical trials.

#### 2.3.3 Subject withdrawal criteria

Subjects will be able to voluntarily withdraw from the study at any time. Upon removal of a subject from the study, laboratory and clinical evaluations that would otherwise have been obtained upon completion of the study will be obtained where possible. In the case of subjects removed due to an AE, the patients will continue to receive medical care and remain under observation by the attending physician for as long as deemed appropriate.

### 2.4 Randomization and allocation

By permuted stratified block randomization (block size 4), patients will be randomly assigned to the control group or intervention group in a ratio of 1:1. Stratification factors will include the type of RT (curative/adjuvant) and the application of concurrent chemotherapy (yes/no). A random number generator based on permutation block design will be used to generate randomized sequences for each stratum separately. The random assignment of each participant will be determined before the intervention. To avoid selection bias, the concealed allocation method will use consecutively numbered opaque sealed envelopes.

### 2.5 Procedure

Both groups will receive conventional cancer treatment, including surgery and RT, with or without chemotherapy based on their disease status. All the subjects in the study will receive RT of 6–10 MV photons delivered by a commercialized linear accelerator with the intensity-modulated radiotherapy or volumetric-modulated radiotherapy technique. The radiation protocol (either definitive or postoperative) will be conventional RT, i.e., 5 fractions per week at 60–70 Gy cumulative doses over 6–7 weeks with or without cisplatin (30–50 mg/m^2^ weekly during radiation) as a radio-sensitizer.

### 2.6 Interventions

The TCM preparation consists of concentrated extract powders manufactured by certified pharmaceutical companies. ZYLGS is recommended by the National Research Institute of Chinese Medicine, Ministry of Health and Welfare, Taiwan, for the treatment of a syndrome with impairment of *qi*/yin by excessive heat, which is characterized by OM during RT. The constituents, proportions, actions, and possible side effects in the Taiwan Herbal Pharmacopeia [15] of each CHM regimen are listed in Table 1. The participants will be instructed to take ZYLGS in doses of 4 g (per bag) each time, thrice daily, from the first day of RT until the completion of RT. All of the drugs will be uniformly packed and stored in a Chinese Medicine pharmacy. For administration, 4 g of the formula will be put into the same container with 100 mL of warm water until the particles are completely dissolved, and then an appropriate amount of water will be added for dilution. Participants in the non-TCM treatment group will receive standard oral care for OM based on the Mucositis Research Group of the Multinational Cancer Support Therapy Association and the International Society of Oral Oncology (MASCC/ISOO). Such care mainly includes brushing with a soft toothbrush, flossing and the use of non-medicated rinses [16].

**Table 1:** Constituents of TCM preparation and their possible side effects.

Pharmaceutical Latin	Pin Yin	Dosage (g/d)	Actions	Possible side effects
*Rx. Scutellariae*	Huang Qin	2	Drains Fire, clears Heat, dries Dampness, detoxifies and clears Lung and Liver Heat.	Gastric discomfort, nausea and vomiting, diuresis
*Rx. Glycyrrhizae*	Gan Cao	2	Moistens the Lungs, resolves Phlegm, stops cough, clears Heat and relieves Fire toxicity.	Edema, high blood pressure, hypokalemia, and arrhythmia
*Hb. Dendrobii*	Shi Hu	3	Nourishes the Yin, clears Heat, generates fluids, enriches Kidney Yin, reduces Heat from Deficiency and nourishes Stomach and Lung Yin.	Allergic dermatitis, seizure
*Rx. Ophiopogonis*	Mai Men Dong	3	Moistens the Lungs, nourishes the Yin, stops cough, nourishes Stomach Yin, generates fluids and moistens the Intestines.	Allergy, palpitation, nausea and vomiting, flatulence, belching, diarrhea
*Hb. Menthae Haplocalycis*	Bo He	2	Disperses Wind-Heat, cools and clears the head and neck, benefits the throat	Heartburn, headache

As noted, patients in the experimental group will be administered medicine in the TCM clinic and receive the TCM preparation from the first week of RT through the end of RT, or for at least 6 weeks during RT. Taking less than 70% of the study medication during the trial period will be considered non-compliance, and patients meeting this criterion will be excluded from the trial. Patients will be evaluated in the oncology and radiotherapy departments every day (except on weekends) for evaluation of mucositis, oral pain, and lesion. Such assessment is possible due to chemotherapy and RT treatment schedule. Patients will record in a diary the manner of the CHM formula administration, schedules, concomitant use with other medications and reports of adverse effects.

### 2.7 Assessments

Each patient will participate in the study for at least 6 weeks, starting from the beginning of RT. Demographic and clinical characteristics recorded at baseline will include gender, age, primary tumor location and stage, scheduled treatment, RT dose, and Karnofsky score. Eligible patients will be randomly assigned to the radiology department for functional assessment and intervention.

The study outcomes will be assessed at baseline (2 weeks pre-RT, t0); at the beginning of RT (t1); at 1 (t2), 2 (t3), 3 (t4), 4 (t5), and 5 (t6) weeks; at the end of RT (t7); and at 2 weeks after completion of RT (t8). The schedule of enrollment is shown in Table 2.

**Table 2:** Summary of collected data at each time point according to SPIRIT 2013 guidelines.

Period week	Enrollment	Allocation	Treatment						Follow-up
	Week −2	Week 0	Week 1	Week 2	Week 3	Week 4	Week 5	Week 6	Week 8
**Enrollment**									
Eligibility screening	x								
Informed consent	x								
Allocation		x							
Baseline information	x								
**Randomization**		x							
**Intervention**									
CHM group			x	x	x	x	x	x	
Control group			x	x	x	x	x	x	
**Assessment**									
OM onset			x	x	x	x	x	x	x
Intensity of OM			x	x	x	x	x	x	x
Nutritional status		x	x	x	x	x	x	x	
Quality of life		x				x		x	
Safety monitoring			x	x	x	x	x	x	x
**Adverse events** (side effects and complications)			x	x	x	x	x	x	x

CHM: Chinese herbal medicine, OM: oral mucositis, TCM: traditional Chinese medicine.

#### 2.7.1 Primary outcome

The primary outcome used in this study will be the onset of OM, intensity of OM and pain. After the start of RT, the patients will be examined daily until onset of OM. Once OM presents, the onset will be recorded in the proforma, and thereafter the degree will be examined weekly until RT is completed and then at 2-weeks post-RT by oncologists.

Subjective assessment of mucositis pain will be performed with a 100-mm visual analog scale (VAS, where 0 = no pain and 100 = worst possible pain) [17], and objective OM grading will be conducted according to the Common Terminology Criteria for Adverse Events, version 5.0 (CTCAE v5.0). The CTCAE v5.0 grades of OM are defined as follows: grade 0, no mucositis; grade 1, asymptomatic or mild symptoms with no intervention indicated; grade 2, moderate pain that does not interfere with oral intake or cause diet adjustment; grade 3, severe pain that interferes with oral administration; grade 4, indication of life-threatening consequences and emergency intervention; and grade 5, death [18].

#### 2.7.2 Secondary outcomes

Secondary outcome measures will include nutritional status and health-related QoL.

The nutritional status evaluation indicators will include body weight, body mass index (BMI), albumin, hemoglobin and total lymphocyte count. Weight change will refer to weight loss during treatment. BMI is calculated by dividing body weight (kg) by the square of height (m). Weight change and BMI will be calculated before and after treatment. Blood indexes will be collected during the weekly routine blood and biochemical examinations.

The EORTC Core Quality of Life questionnaire (EORTC QLQ-C30) contains a series of HR-QoL issues related to a wide range of cancer patients. It has been translated into multiple languages and verified for multiple types of cancer, including HNC. It contains five functional scales (physical, role, cognitive, emotional and social), three symptom scales (fatigue, pain and nausea/vomiting), a global QoL scale, and six individual items (dyspnea, insomnia, appetite loss, constipation, diarrhea, and financial difficulties) [19]. The head and neck module (QLQ-H&N35) is a module used for evaluating the HR-QoL of HNC patients. It contains seven multi-item scales for evaluating pain symptoms, swallowing ability, senses (taste/olfaction), speech, social eating, social contact, and sexual behavior. It also includes six single-item scales to investigate the presence of symptoms related to teeth, mouth opening, dry mouth (xerostomia), sticky saliva, coughing, and feeling ill [20]. All scales of the EORTC QLQ-C30 and QLQ-H&N35 range from 0 to 100. A high score on the functional or global HR-QoL scale represents a relatively high/healthy functional level or global QoL, while a high score on the symptom scale represents the presence of symptoms or problems. Patients will complete both questionnaires before, at the 4th week of, and after RT treatment.

#### 2.7.3 Safety assessments

No serious AEs are anticipated as a result of the study, and the clinicians will monitor the risks. In cases of AEs caused by anti-cancer treatment or CHM intervention, we will promptly handle the AEs or withdraw the participant from the study. A series of measures to evaluate the safety of the ZYLGS will be monitored throughout the entire trial due to possible herb-induced toxicity. The participants’ blood, liver and kidney function, body weight, and vital signs will be monitored weekly during the treatment period. AEs will be categorized according to the Patient-Reported Outcomes version of the Common Terminology Criteria for Adverse Events (PRO-CTCAE ITEMS Version 1.0) [21] and recorded throughout the study.

For monitoring of the AEs or serious AEs, an independent Data and Safety Monitoring Board (DSMB) review will be conducted for every 50 patients participating in the trial, for a total of four DSMB meetings. A comparative analysis of the incidence of AEs based on the intervention group will be performed at each DSMB review. The DSMB will make recommendations to the research team on appropriate action plans to resolve research safety issues that may arise during the trial. Only when it is determined that an intervention is absolutely necessary for further management of the participants, such as in the event of SAEs, will the research team be un-blinded.

### 2.8 Statistical analysis

#### 2.8.1 Sample size

The sample size estimation was based on the mean difference in the change of the OM intensity from baseline. Based on a previous study, we made the following assumptions in calculating the sample size: a mean difference of 0.9 between the two groups, SD of total score of 0.7, a ratio of 1:1 between the two groups, a two-sided significance level of 0.025 and a power of 0.9 [10]. With these assumptions, a minimum sample size of 34 patients (17 patients in each group) is needed. Considering the estimated 15% dropout rate, the estimated sample size is 40 patients (20 patients per group).

#### 2.8.2 Data analyses

All statistical analyses will be performed by qualified statisticians in SPSS software (SPSS Inc., Chicago, IL, USA; version 13.0) based on the intention to treat (ITT) principle. The data will also be collected and analyzed according to said principle. Continuous variables will be expressed as mean ± SD and compared using unpaired independent *t* tests. Chi-square tests will be used to compare the categorical data between groups.

A two-sided *p* < 0.05 will be considered significant. The trends of change over time in degree of OM, each scale/item of the EORTC QLQ-H&N35 and EORTC QLQ-C30, will be compared between groups with the generalized estimating equation (GEE) in SAS software version 9.4.

#### 2.8.3 Data collection and management

All data will be recorded on the case report form (CRF) and stored anonymously in laboratory computer at the KCGMH. Investigators will provide access to data files based on reasonable requests. The researcher is ultimately responsible for collecting and reporting all applicable data entered into the CRF in a timely manner, and ensuring that they are accurate, original, attributable, complete, legible, and available when needed.

## 3 Discussion

RT-induced OM is a common problem and one of the major causes of pain and functional incapacity in HNC patients [3]. The present study aims to assess the effects of our TCM preparation on prevention and management of the pain, severity, and QoL of HNC patients with OM.

Many CHMs show significant improvements in side effects related to anti-cancer treatment, and TCM is the most common means of CIM in Taiwan. In a previous nationwide cohort study in Taiwan, 12.6% of patients with HNC used CIM, and the authors found that the number of TCM visits increased during multiple cancer treatments [22]. Another study reported the utilization of TCM to improve the survival outcome for HNC, and the results showed that longer use was associated with lower mortality [23]. We believe that it is possible to select formulae based on the pathogenesis of RT-induced OM and to use TCM theory to reduce the side effects of cancer treatment.

According to TCM theory, RT is a type of heat toxin that damages *qi* and yin and gradually affects the patient's physical strength [13]. Chemotherapy is also considered to aggravate a lack of *qi* [24], which can make wound healing more difficult [25]. ZYLGS provides an attractive therapeutic option for the prevention and management of OM, for the concept of using herbal components to clean the heat, replenish *qi* and nourish yin has been recorded in classical TCM books. Modern pharmacological research shows that *Rx. Scutellariae*, *Rx. Glycyrrhizae* and *Hb. Menthae Haplocalycis* have positive effects on oral epithelial damage. Their mechanism may be to inhibit oxidative stress and inflammation and thereby prevent bacterial dysbiosis [14]. *Hb. Dendrobii* can modulate macrophage activity via the NF-kB pathway and has been shown to act as an immune modulator to regulate gastrointestinal mucosal barrier function [26,27]. *Rx. Ophiopogonis* has been reported to stimulate lymphocyte proliferation *in vitro* [28]. Both are used for the benefit of *qi* and to promote body fluid production, according to TCM theory, and they are also reported to have the effects of cardiovascular protection, anti-inflammation and anti-cancer [27,29,30]. Although *in vitro* and *in vivo* studies have provided evidence supporting the purported effects of these ingredients of ZYLGS, we still perceive a need for a clinical trial to assess the efficacy of CHM formula in treating RT-induced OM.

Each medication in the ZYLGS formula is described in the ancient TCM literature as relatively safe, and in previous meta-analyses, therapeutic doses have been found to cause no significant toxicity in cancer patients [31,32]. However, some serious adverse reactions have been reported from time to time in recent years. For example, it has been reported that prolonged use or overdose of *Rx. Glycyrrhizae* leads to pseudohyperaldosteronism such as hypertension, hypokalemia or metabolic alkalosis, with low serum aldosterone levels [33]. For potential immunoallergic reactions, such as those associated with *Rx. Scutellariae*, which has liver [34] and lung toxicity [35], attention should also be paid to the safe range of medicine usage, the dosage should not be excessive, and the processed products should be selected appropriately according to needs. Despite their sourcing from TCM pharmaceutical factories in Taiwan with Good Manufacturing Practices certification, the safety and herb–drug interactions and toxicity must be monitored.

Our study has some limitations. One is that the research is being conducted in a single center with a small sample size. Second, the study is not a double-blind trial. Third, only short-term observations can be made, so this study cannot produce a long-term prognosis. For example, it will not be possible to predict whether the reduction of side effects of CHM formula will affect the RT cytotoxic effect and thus affect the survival rate.

In conclusion, we have proposed a randomized controlled, open-label, single-center trial to evaluate the efficacy and safety of ZYLGS as a CIM therapy for RT-induced OM. The results of the current research will provide high-quality evidence for assessment of the prophylaxis of RT-induced OM using a TCM-based prescription of ZYLGS.

## Funding

This study is funded by the 10.13039/100008903National Research Institute of Chinese Medicine, Ministry of Health and Welfare, as part of the Featured Areas Research Center Program in Taiwan (MOHW110-NRICM-M-124-112004 & MOHW111-NRICM-M-124-122004). The institute provided financial support and reviewed the study design at the time of approval, but it has had and will have no involvement in the collection, analysis, and interpretation of data or writing of the manuscript.

## Authors’ contributions

Study conception and design: YMW, SHL, YHL, JYC, MYT. Data analysis planning: YCC, YCH, MYT. Drafting of the manuscript: YMW, SYC, MYT. Critical revisions: all authors. Final approval of the article: all authors. Obtaining funding: MYT, who is responsible for the integrity of the work as a whole. YCC and YMW will contribute equally to this study.

## Trial registration

Clinicaltrials.gov registry (NCT05040425), registered 03 September 2021; Pre-results.

## Declaration of competing interest

None declared.

## Data Availability

Data will be made available on request.

## List of abbreviations

AE: adverse event
ALT: alanine aminotransferase
AST: aspartate transaminase
BCQ: Body Constitution Questionnaire
BMI: body mass index
CCRT: concurrent chemo-radiotherapy
CHM: Chinese herbal medicine
CIM: complementary and integrative medicine
CRF: case report form
CTCAE: Common Terminology Criteria for Adverse Events
DSMB: Data and Safety Monitoring Board
ECOG: Eastern Cooperative Oncology Group
EORTC QLQ-C30: EORTC Core Quality of Life questionnaire
GEE: generalized estimating equation
HNC: head and neck cancer
ITT: intention to treat
OM: oral mucositis
QLQ-H&N35: Quality of Life Head and Neck Module
QoL: quality of life
RT: radiotherapy
TCM: traditional Chinese medicine
ZYLGS: Zi-Yin-Liang-Ge-San
